# Treatment of Ulcers of the Leg by Scabbing

**Published:** 1897-06-05

**Authors:** 


### TREATMENT OF ULCERS OF THE LEG BY SCABBING.

Professor Colville, of the Rheims School of Medicine, has introduced a method of treating ulcers of the leg by light, heat, and ventilation, which he claims to have been very successful. The skin round the ulcer is protected by compresses, and then a small square of wire gauze, kept at a red heat by a Bunsen burner, is placed at about ten inches distance. Free ventilation around is maintained all the time. A temperature of about 45 deg. C. is maintained for 20 minutes, when the surface of the ulcer is found to be covered with a layer of glazed solidified serum, through which the granulation tissue can be distinctly seen. The leg is then bandaged, a hole being left opposite the ulcer. This hole is then covered in with aseptic gauze, which, however, must not touch the ulcer, which is thus left dry. The whole process would appear to be a systematised endeavour to apply the principle of healing under a scab to wounds which have already suppurated. The scab formed under ordinary haphazard conditions usually becomes septic, and thus the healing process is interfered with; but we can quite imagine that a scab rapidly formed under the influence of heat, from serum freshly effused, may form a very efficient and unirritating protective layer under which healing may take place.

